# An improved method for gastric tube and anastomotic anvil placement during thoracoscopic and laparoscopic Ivor Lewis esophagectomy

**DOI:** 10.1186/s12957-020-01892-x

**Published:** 2020-05-28

**Authors:** Yi Shen, Yunfeng Zhou, Xiang Zhuang

**Affiliations:** 1 Department of Thoracic Surgery, West China School of Public Health and West China Fourth Hospital, Sichuan University, Chengdu, 610041 China; 2 Department of Thoracic Surgery, Sichuan Cancer Hospital, School of Medicine, University of Electronic Science and Technology of China, Chengdu, 610041 China

## Abstract

**Background:**

During esophagectomy for esophageal cancer, a gastric tube is necessary for the perioperative period. However, the gastric tube and anastomotic anvil placement is often extremely difficult and time consuming during surgery.

**Methods:**

We used the traditional method or improved method to place the gastric tube and anastomotic anvil during thoracoscopic and laparoscopic Ivor Lewis esophagectomy. Thirty-seven patients were in the improved group: the gastric tube and anastomotic anvil were placed using the improved method; 35 patients were in the traditional group: the gastric tube and anastomotic anvil were placed using the traditional method. Retrospectively, we analyze the basic clinical characteristics, perioperative clinical features, and postoperative complications of the two groups of patients.

**Results:**

The two groups were matched well for baseline characteristics. There was no significant difference between the two groups in blood loss, postoperative hospital stay, postoperative fasting time, drainage volume, and overall complications. But significant between-group differences were observed in time consuming and chest tube indwelling time (*P* < 0.05), both of which were significantly shorter in the improved group than in the traditional group.

**Conclusions:**

This improved method can reduce the difficulty of placing anastomotic anvil and gastric tube and prevent damage to the anastomosis during surgery.

## Introduction

During esophagectomy for esophageal cancer, a gastric tube can be placed for gastrointestinal decompression, alleviation of postoperative abdominal distention, reduction of anastomotic pressure, and observation of anastomotic bleeding. In thoracoscopic and laparoscopic Ivor Lewis esophagectomy, a gastric tube is typically placed in the patient through the nasal cavity after anastomosis. However, due to uncertainty regarding the direction of the gastric tube tip, tube placement is often extremely difficult, requires a lengthy duration, and can even cause damage to the anastomosis. Additionally, when the anastomotic anvil is placed prior to anastomosis, there is no directional traction during delivery of the anastomotic anvil and the operation is often time consuming. Moreover, violent delivery of the anvil may also lead to anastomotic damage. Using the technique described here, we have improved the method of anastomotic anvil and gastric tube placement to reduce not only the placement duration but also the incidence of anastomotic damage.

## Technique

### Laparoscopic phase

The patient was placed in a supine position. A total of five abdominal ports were used, including two 5-mm ports, two 10-mm ports, and one 12-mm port. An artificial pneumoperitoneum was established using CO_2_. Perigastric tissue was transected, and the abdominal lymph nodes were cleaned. A tubular stomach was formed using a straight cutting stapler under laparoscopy.

### Thoracoscopic phase

The patient was placed in the left lateral decubitus position. A total of four ports were established, including a 10-mm observation port (at the seventh intercostal space along the midaxillary line), a 40-mm main operation port (at the fourth intercostal space along the anterior axillary line), a 5-mm auxiliary port (at the seventh intercostal space along the infrascapular line), and a 10-mm suboperation port (at the ninth intercostal space along the posterior axillary line). The thoracic esophagus was dissociated, and the mediastinal lymph nodes were cleaned. The esophagus was transected 6 cm from the top edge of the tumor. The tumor and part of the gastric tissue were extracted.

### Placement of the gastric tube and anastomotic anvil

**Fig. 1:**
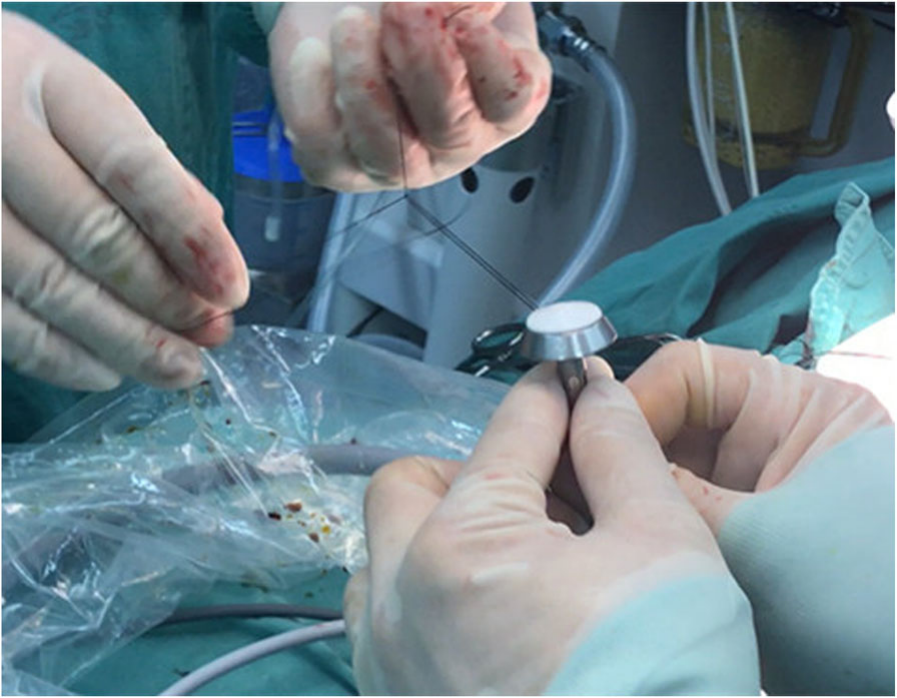
A thread was ligated to the top of the mushroom head of the anastomotic anvil

**Fig. 2:**
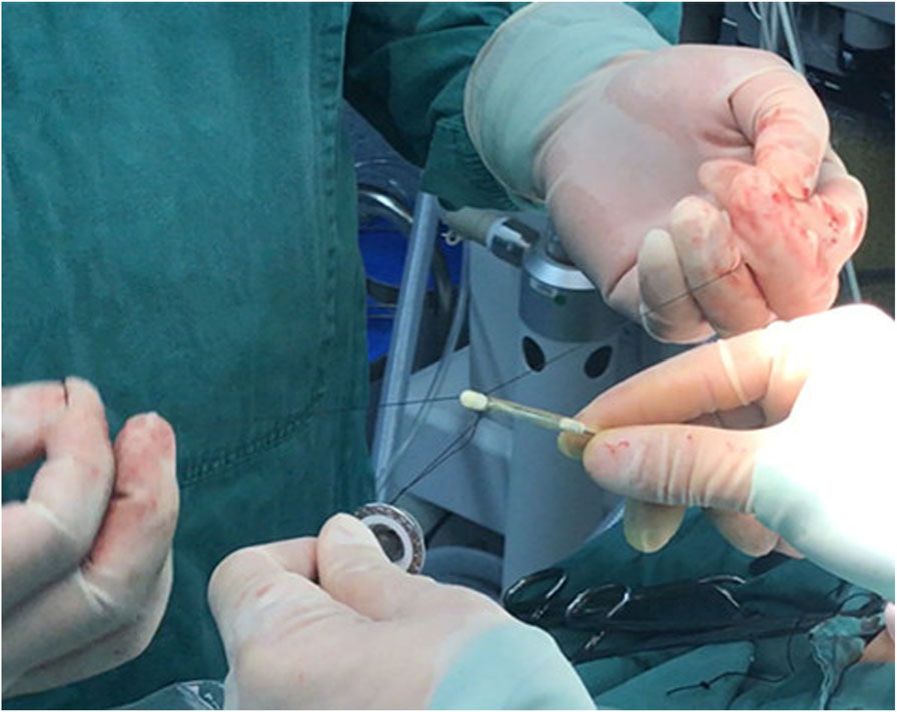
Anastomotic anvil and gastric tube were connected using the threads

**Fig. 3:**
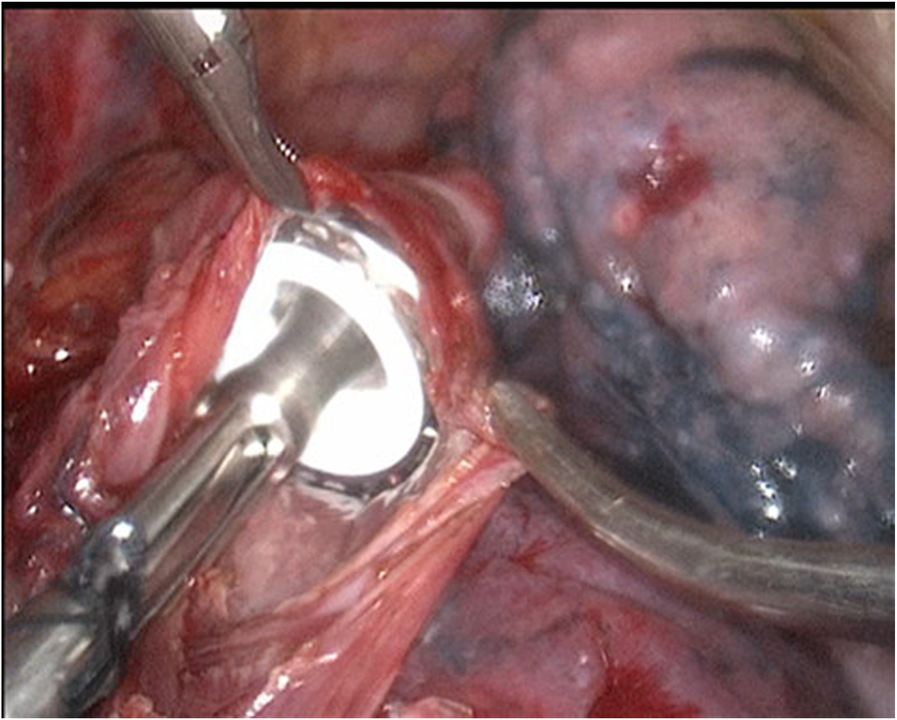
The anastomotic anvil was pulled to the anastomotic area

(1) A no. 7 thread that would be used to pull the anastomotic anvil was ligated to the top of the mushroom head of the anastomotic anvil (Fig. 1). (2) The gastric tube was placed into the esophagus through the nasal cavity, the stomach tube was drag out of the chest cavity through the esophagus stump, and the anastomotic anvil and gastric tube were connected using the threads (Fig. 2). (3) The anastomotic anvil was pulled to the anastomotic area of the esophageal stump by pulling the gastric tube (Fig. 3). (4) Esophagogastric side-to-side anastomosis was performed using reverse-puncture anastomotic technique [1]. (5) Pull the tip of the gastric tube near the anastomosis by pulling the anastomotic anvil. The traction thread at the tip of the gastric tube was cut, and the anastomotic anvil was removed. (6) Put the tip of the gastric tube into the tubular stomach through the anastomosis, and push the gastric tube to an appropriate position in the stomach. The anastomosis was completed by closing the gastric stump using a stapler. The operation ports were closed after placement of a thoracic drainage tube and a mediastinal drainage tube at the observation port and the port at the ninth intercostal space, respectively.

## Results

The clinical data of 72 patients undergoing Ivor Lewis esophagectomy were retrospectively analyzed. Thirty-seven patients were in the improved group, the gastric tube and anastomotic anvil were placed using the above method, while the traditional group was using the traditional method.

The two groups were matched well for baseline characteristics including sex, age, history of smoking and drinking, tumor location, pathologic stage, histological type, and comorbidities (Table 1).

**Table 1:** Basic clinical characteristics

Characteristics	Improved group (*n* = 37)	Traditional group (*n* = 35)	*P* value
Age (years)	56.5 ± 10.2	53.9 ± 9.7	0.279
Gender (male/female)	25/12	24/11	0.927
Smoking	27	21	0.243
Drinking	18	16	0.803
Tumor location (middle/lower)	6/31	9/26	0.321
Pathologic stage			
I	11	8	0.445
II	19	23	
III	7	4	
Histological type			
Squamous cell	32	33	0.472
Adenocarcinoma	5	2	
Comorbidities^#^	3	6	0.422

^#^Hypertension, diabetes, etc.

There was no significant difference between the two groups in blood loss, postoperative hospital stay, postoperative fasting time, and drainage volume. But significant between-group differences were observed in time consuming and chest tube indwelling time (*P* < 0.05), both of which were significantly shorter in the improved group than in the traditional group (Table 2).

**Table 2:** Perioperative clinical features between the two groups

Clinical features	Improved group (*n* = 37)	Traditional group (*n* = 35)	*P* value
Time consuming^#^ (min)	21.3 ± 2.7	24.8 ± 3.1	0.000
Blood loss (ml)	337.6 ± 137.0	302.7 ± 138.2	0.286
Postoperative hospital stay (days)	11.1 ± 2.6	11.7 ± 3.1	0.254
Chest tube stay (days)	4.4 ± 1.8	5.5 ± 3.4	0.023
Postoperative fasting time (days)	6.6 ± 2.0	7.1 ± 3.2	0.326
Drainage volume (ml)	1122.3 ± 228.1	1047.6 ± 221.4	0.163

^#^Gastric tube placement and esophagogastric anastomosis time consuming

There was no significant difference between the two groups in the overall complication rate, the incidence of anastomotic leakage, pulmonary infection, arrhythmia, wound infection, or recurrent laryngeal nerve palsy (Table 3).

**Table 3:** Postoperative complication between the two groups

Complications	Improved group (*n* = 37)	Traditional group (*n* = 35)	*P* value
Total complications	7 (18.9%)	9 (25.7%)	0.488
Leakage from anastomosis	1 (2.7%)	4 (11.4%)	0.321
Pneumonia	4 (10.8%)	3 (8.6%)	1.000
Arrhythmia	1 (2.7%)	2 (5.7%)	0.961
Chylous fistula	0	1 (2.9%)	1.000
Recurrent laryngeal nerve paralysis	2 (5.4%)	1 (2.9%)	1.000

## Discussion

Shortly after esophagectomy, patients often exhibit delayed gastric emptying, gas and secretions in the gastrointestinal tract, and occasional bleeding at the anastomosis or the gastric margin, leading to the accumulation of gastrointestinal contents, which causes gastric dilatation [2]. Gastric dilatation increases tension at the anastomosis, increasing the incidence of anastomotic leakage. Moreover, increased stomach tension may also lead to vomiting, which increases the risk of aspiration pneumonia [3]. Therefore, it is extremely important to place a gastric tube after esophagectomy and induce early gastrointestinal decompression [4] such that gas and secretions can be removed in a timely manner, thereby reducing tension in the stomach and at the anastomosis, ameliorating the corrosive effect of gastric acid on the anastomosis, and enabling the detection of bleeding of the surgical wound.

This study shows that the incidence of anastomotic leakage in the traditional group is significantly higher than that in the improved group, but the difference is not statistically significant, which may be related to the small sample size.

In terms of placement time, the improved group was significantly shortened. The modified group did not increase surgical trauma and operation time; therefore, the improved method had no effect on postoperative lung infection and recurrent laryngeal nerve injury; just as this study, there is no statistically significant difference in complications.

Traditionally, the gastric tube has been placed blindly after completion of the anastomosis. Since it is often difficult for the tip of the gastric tube to pass through the relatively narrow anastomotic region, tube placement can cause anastomotic damage and may lead to postoperative anastomotic leakage. During prior Ivor Lewis procedures, when placing the mushroom head of the anastomotic anvil, the surgeon would position the head into the esophageal anastomotic area by clamping the center rod with forceps. A thoracoscopic operation requires skilled surgical techniques, and improper operations are often lengthy and can even cause mucosal damage in the anastomotic area.

Therefore, the improved method that we used for placing the gastric tube and anastomotic anvil has the following advantages. (1) The anastomotic anvil can be smoothly and rapidly placed into the anastomotic area by pulling the gastric tube, and improper operation-induced damage to the anastomosis can be prevented. (2) After completion of the anastomosis, the gastric tube can be smoothly pulled underneath the anastomotic stoma by pulling the anastomotic anvil, shortening the gastric tube placement time and preventing injury to the anastomotic stoma caused by the tip of the gastric tube. However, this method has many steps, and at the same time, the gastric tube is pulled out of the esophagus stump to increase the risk of infection. This method needs to be further improved.

In summary, placing the gastric tube and anastomotic anvil using the pulling method not only is simple, easy to learn, and safe but also allows for rapid operations; thus, this approach merits widespread application in clinical practice.

## Data Availability

As a technical innovation, all data generated or analyzed are included in this published article.
